# High energy X-ray phase and dark-field imaging using a random absorption mask

**DOI:** 10.1038/srep30581

**Published:** 2016-07-28

**Authors:** Hongchang Wang, Yogesh Kashyap, Biao Cai, Kawal Sawhney

**Affiliations:** 1 Diamond Light Source Ltd, Harwell Science and Innovation Campus, Didcot, OX11 0DE, UK; 2 School of Materials, University of Manchester, Manchester, M13 9PL, UK; 3 Research Complex at Harwell, Rutherford Appleton Laboratory, Harwell, Oxfordshire, OX11 0FA, UK

## Abstract

High energy X-ray imaging has unique advantage over conventional X-ray imaging, since it enables higher penetration into materials with significantly reduced radiation damage. However, the absorption contrast in high energy region is considerably low due to the reduced X-ray absorption cross section for most materials. Even though the X-ray phase and dark-field imaging techniques can provide substantially increased contrast and complementary information, fabricating dedicated optics for high energies still remain a challenge. To address this issue, we present an alternative X-ray imaging approach to produce transmission, phase and scattering signals at high X-ray energies by using a random absorption mask. Importantly, in addition to the synchrotron radiation source, this approach has been demonstrated for practical imaging application with a laboratory-based microfocus X-ray source. This new imaging method could be potentially useful for studying thick samples or heavy materials for advanced research in materials science.

X-rays imaging has experienced golden age following Röntgen’s discovery of X-rays, and it proves to be a powerful and invaluable tool for many fields, such as, medical diagnosis, material science, archaeology, food industry and security screening. Within the medium X-ray range (10–30 keV), absorption contrast is normally low for light materials (made up of low-Z elements), such as soft tissues, carbon composites, etc. On the contrary, substantially enhanced contrast can be observed in the corresponding phase signal. In addition, X-ray dark-field imaging can further distinguish fine details and subtle differences from scattering information within the sample. Unlike absorption contrast imaging, both X-ray phase and dark-field contrast images cannot be measured directly; either sophisticated experimental conditions or stringent beam properties are usually required. Even though X-ray phase contrast imaging was first demonstrated in 1965 1, it wasn’t until mid-nineties that it started booming, thanks to the availability of highly brilliant synchrotron radiation sources and the advanced X-ray optics. In parallel, dark-field imaging has been realized in the X-ray regime and provides the scattering information of sub-pixel microstructure within a sample^2^. In order to investigate thick samples or high-density materials (made up of high-Z elements), it is imperative to apply X-ray imaging at high energies (over 100 keV). As expected, the absorption contrast for materials with similar atomic number is low due to a strongly reduced absorption cross section at high X-ray energies. It should be pointed out that the cross section between coherent scattering and absorption is similar to each other for high energy X-rays, hence even higher sensitivity is required for phase sensing techniques. Additionally, it becomes more challenging to detect the dark-field signal since the scattering power also reduces dramatically with increased X-ray energy. In the last several years, a few X-ray phase contrast imaging techniques have been extended up to 85 keV^3^,^4^,^5^,^6^, however the fabrication of precision optics for high energy X-ray imaging still remains a challenge.

Recently, the X-ray shearing interferometry has been demonstrated to push the working energy over 100 keV^7^,^8^. As shown in Fig. 1a, the grating interferometer normally consists of a first phase grating as a beam splitter and a second absorption grating that acts as a transmission mask. One of the main technical challenges here is to fabricate the grating structures with extremely high aspect ratio (defined as *h*/*w* in Fig. 1a) in order to produce sufficient phase shift (for phase grating) or to block the high energy X-rays (for absorption grating). For instance, the required height (*h*) of the grating rulings has to be around 1 mm in order to absorb most of the incoming X-rays at energy of 100 keV by using gold as absorption material. To overcome this limitation, another approach, as illustrated in Fig. 1b, has been proposed to shine X-ray along the grating line direction^9^. Although a high aspect ratio can be achieved, the narrow field of view still limits its use for practical application. Over the last few years, the speckle-based technique have shown tremendous potential for X-ray multimodal imaging by simply utilizing a speckle generator (such as, a sheet of abrasive paper)^10^,^11^,^12^,^13^,^14^,^15^,^16^; and it usually requires moderate transverse coherence to generate these speckle patterns. For synchrotron radiation X-ray sources, the phase contrast speckle can be observed in the near field region if the average grain size is comparable the transverse coherence length. The requirement for the grain size of abrasive paper turns out to be more stringent for a microfocus X-ray source with relatively lower transverse coherence. In order to resolve the phase contrast speckle pattern, it often requires a high resolution detector, which inevitably suffers from smaller field of view and longer data acquisition^17^,^18^.

In the medium X-ray energy regime, instead of using the phase contrast speckle pattern, it is possible to observe the absorption based random pattern from the abrasive paper^13^. Nevertheless, the absorption contrast is poor since the abrasive paper commonly consists of light abrasive materials, such as silicon carbide or aluminium oxide. As shown in Fig. 1c, the visibility *V*, defined as the ratio of the standard deviation to mean of the absorption based random pattern, is too low (*V* ≈ 3%) to be useful for imaging at higher energies. To circumvent above limitations, we propose an alternative X-ray phase and dark-field imaging approach for high energy material inspection by using a random absorption mask.

It may be noted that the concept of the proposed approach is in a way bears some similarity with the edge illumination technique^19^,^20^, wherein a precisely fabricated gold mask was used in conjunction with the beam tracking approach to obtain the multimodal images. In contrast, in this study, instead of using the precise gold mask, high precision grating or abrasive paper, an absorption mask consisting of bundle of randomized and uniformly distributed high Z material is employed to generate a high absorption contrast pattern. Here, steel wool was chosen as absorption material since it is commercially available and a wide of range of fibre thickness (from 25 μm up to 100 μm) can be selected based on the experimental requirement. However, we would like to emphasize that the use of steel wool as absorption mask is not as straightforward as the abrasive paper. As shown in Fig. 1d, the random pattern with higher absorption contrast (*V* ≈ 15%) can be generated by placing the random collection of steel wool perpendicular to the X-ray direction. However, as highlighted in the Fig. 1d with red circle, some large empty spaces exist due to the non-uniform distribution of the steel wool. Such gaps can also be seen in the intensity profile along the marked black line in the Fig. 1d. Since these empty spaces don’t have random absorption structures, some artefacts will inevitably get introduced for speckle scanning technique. Therefore, we propose to place the steel wool strands along the X-ray direction. In addition, the steel wool strands were cut and re-mixed properly to ensure that new absorption mask creates uniform and random structure over the illuminated area. As shown in Fig. 1e, the generated randomly distributed pattern is similar to the one produced by an abrasive paper, but with a much higher visibility of *V* ≈ 17%. Compared to the line profile shown in Fig. 1d, more oscillations can be observed in the intensity profiles along the marked green line in the Fig. 1e. Moreover, the visibility can be further improved by adjusting the length of the steel wool and changing the inter-strand distance in the steel wool. In this letter, we demonstrate that high quality transmission, phase and dark-field images can be obtained by using the proposed X-ray imaging concept for high X-ray energies. Additionally, we also show that this technique can be used with high brilliant synchrotron radiation source as well as laboratory-based microfocus X-ray source.

As described above, the random patterns in the X-ray images are solely produced due to the absorption contrast. Similar to the speckle pattern, the inherent high frequency features of generated random pattern allow them to be used as beam markers. Once the sample is moved into the beam, the reference pattern is shifted and distorted due to the phase gradients and scattering induced by the sample^14^,^15^. The principle of this technique is to analyze the displacement and degradation of random patterns with and without sample in the X-ray beam. It should be pointed out that the deflection angle induced by the sample is generally very small (a few microradians), and therefore the corresponding displacement is only a few micrometres in the detector plane. Although it requires only a single image for the speckle tracking mode^10^,^11^, both the spatial and angular resolutions are compromised by selecting a surrounding subset area in order to perform the cross-correlation algorithm. These limitations can be overcome by raster scanning the speckle generator (such as, abrasive paper) along two orthogonal directions. However, it is too time-consuming to apply the two-dimensional (2D) raster scanning for practical applications^12^,^17^. Therefore, we use the recently developed one-dimensional (1D) scanning mode for the high energy X-ray imaging^13^. The absorption mask, consisting of steel wool strands, is scanned transverse to the beam (without sample present) along the vertical direction in this study. The procedure is then repeated with the sample inserted into the beam. Thanks to the 2D nature of the random pattern, the recorded pattern translates not only along vertical direction but also along horizontal direction after inserting the sample. For each individual pixel, in addition to the signal along the scan direction, a few nearby pixels along horizontal are also selected to trace the random pattern displacement in horizontal direction. The cross-correlation algorithm is then applied between the signal recorded in absence *f* and presence *g* of the sample at each detector pixel (*x*, *y*)^21^ to derive quantitative information. In order to precisely find the maximum of the cross-correlation coefficients, sub-pixel registration algorithm, such as Newton-Raphson method, the curve-fitting method, can be used^22^. Although higher accuracy can be potential achieved by using Newton-Raphson method, the 2^nd^ order polymer curve-fitting is employed in this study due to its simplicity and speed. The located maximum position can be described with the coordinate 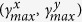 and the magnitude (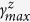). Hence, the coordinate 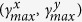 is corresponding to the horizontal and vertical speckle displacement. Following a basic geometric relationship, the wavefront gradient *α*^*x*^, *α*^*y*^ along the horizontal and vertical directions can be directly calculated from the corresponding speckle displacement^13^


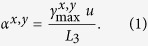


Here *u* is detector’s pixel size *P* for horizontal direction, while it is defined as *u* = *μ*(*L*_1_ + *L*_2_ + *L*_3_)/*L*_1_ for vertical scan direction with the scanning step size of *μ*. Distances between the source, absorption mask, sample and detector are represented by*L*_1_, *L*_2_ and *L*_3_. The first derivative of the sample’s phase shift ∇Φ can be directly calculated from the wavefront gradients. As described in ref. 23, the complex field *g* can then be defined from the two transverse phase gradient, and the two dimensional Fourier transform (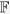) of the complex field is expressed as 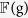. According to the Fourier derivative theorem, and the wavefront phase shift Φ induced by the sample can then be reconstructed by further applying inverse Fourier transform (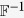) of the term 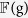
23, where 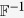 (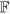) is the inverse (forward) Fourier operations.

After the speckle displacement has been retrieved, the new signals *f*′ and *g*′ can be generated by compensating the corresponding displacement. It should be noted that a median filter is required to apply to the speckle displacement map in order to remove the artefacts at sharp edges [see Supplemental Material]. The transmission *T* is defined as the ratio of the average of two new signals *f*′ and *g*′ collected in each pixel^13^,^16^.


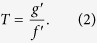


The dark-field signal *D*, related to internal structure inhomogeneity, or electron density variations, can be defined as the second moment of the scattering angle distribution^24^,^25^,^26^. As deduced in previous work, the relation between dark-field and the maximum of correlation coefficient 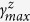
can be quantitatively written as^13^:


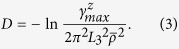


Here, the average spatial frequency 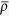 is related to average structure 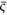 of the absorption pattern as 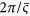.

Therefore, two orthogonal wavefront gradients, phase image, dark-field image and transmission image can simultaneously be acquired whilst translating the random absorption mask in only one direction.

## Results

**Figure 2:**
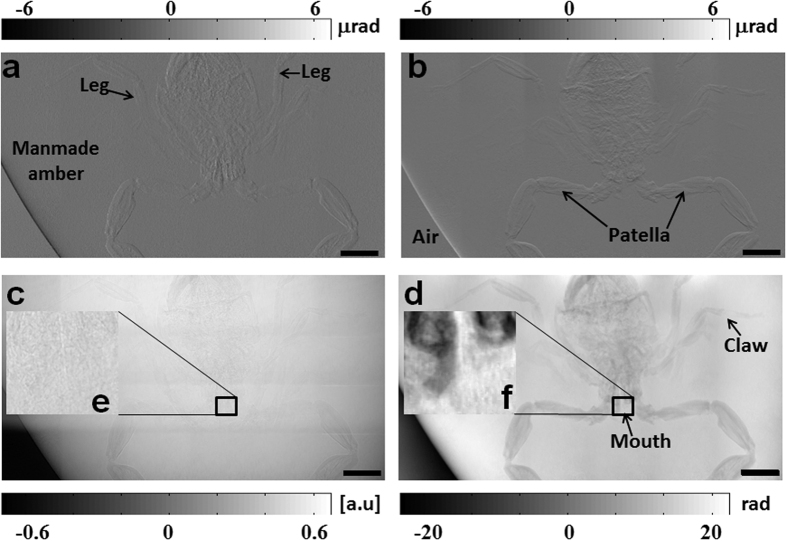
Retrieved differential wavefront gradient, transmission and phase image of manmade amber by using synchrotron radiation source. (**a**) Horizontal and vertical (**b**) differential wavefront gradient, (**c**) absorption and (**d**) reconstructed phase images of a scorpion inside of manmade amber. (**e**) and (**f**) are 10 times zoom in image of the marked regions in (**c**) and (**d**) respectively. The scale bar is equal to 2 mm.

The capability of the proposed technique for non-destructive sample investigation was first validated with a scorpion sample enclosed within manmade amber by using the synchrotron radiation source at Diamond Light Source’s B16 Test beamline^27^. A sub-pixel registration cross-correlation algorithm was used to obtain the cross-correlation map in real space^22^. Following the above procedure, pixel-wise analysis was then performed to extract transmission, horizontal and vertical differential phase images as well as reconstructed phase image. Although high energy X-ray permit the inspection of thick sample, the absorption cross sections for both manmade amber and scorpion samples are significantly reduced. As shown in Fig. 2c, it is difficult to observe the scorpion structure in the conventional transmission image. In contrast to transmission image, the marked legs can be clearly seen in the horizontal wavefront gradient image (Fig. 2a), while they are hardly noticeable in the vertical one (Fig. 2b). In addition, the horizontal patella can be clearly observed in Fig. 2b, but is barely detectable in the horizontal gradient image (Fig. 2a) and vice versa. Hence the two directional differential contrast images provide complementary information, especially for the anisotropic features. To evaluate the angular sensitivity for the two orthogonal wavefront gradients^22^,^23^, the standard deviation of wavefront gradients in empty space (air) are calculated with 300 × 300 detector pixels. For the present experimental geometry, the angular sensitivity is about 200 nrad and 80 nrad for the horizontal and vertical direction, respectively. The phase image of the sample is reconstructed from the two orthogonal phase gradient images. Unlike the conventional transmission image (Fig. 2c), enhanced contrast in scorpion sample can clearly be observed in the phase image (Fig. 2d). As highlighted in the rectangle region, the scorpion mouth is hardly noticeable in the absorption contrast image, while subtle structures can be distinctly observed in the phase contrast image. To quantitatively describe the contrast of the absorption and phase images, we use contrast radio (*CR*), which was calculated by the relation: *CR* = σ(*S*)/σ(*R*). Here σ(*S*) and σ(*R*) is the standard deviation in the selected area around sample (scorpion mouth) and reference area (manmade amber). For the above highlighted sample region (400 × 400 pixels), the calculated values of CR for absorption and phase are found to be 3.0 and 5.9, respectively. In order to retrieve both horizontal and vertical speckle displacement in the pixel wise analysis process, two neighbouring pixels were selected. Hence, the effective spatial resolution (11.4 μm) of the image is about three time of the detector pixel size for both absorption and phase images. This example shows that it is beneficial to use the X-ray phase contrast imaging in the higher energy region to reveal subtle details from the samples with better contrast ratio. Importantly, different contrast images can provide complementary information for comprehensive investigation, and all the different images are extracted simultaneously from a single data.

**Figure 3:**
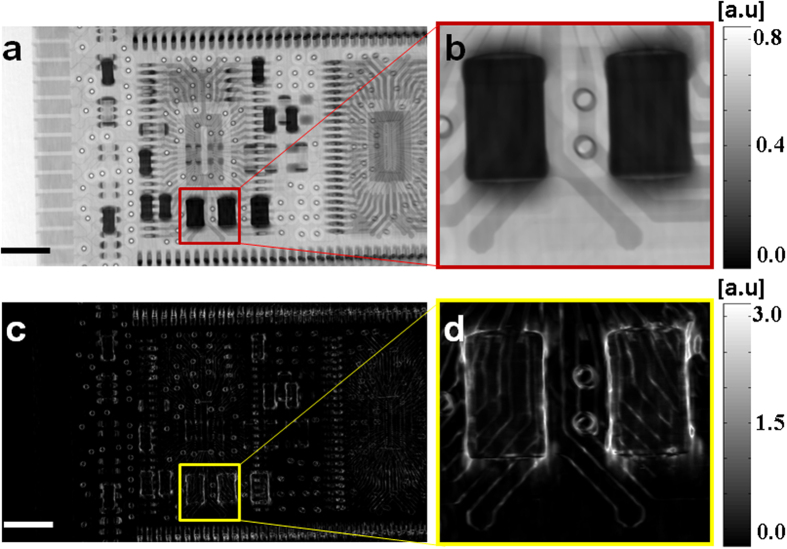
Retrieved transmission and dark-field image of a microchip by using laboratory X-ray microfocus source. (**a**) Transmission and (**c**) dark-field image, (**b**,**d**) are the magnified parts from image (**a**,**c**). The scale bar is equal to 2 mm.

To demonstrate the applicability of the presented X-ray imaging technique for potential practical applications at high X-ray energies, further experiments were carried out with a laboratory X-ray microfocus source. Since the X-ray flux from the microfocus source is considerately lower compared to the synchrotron radiation source, it is even more challenging for practical X-ray imaging applications by using high resolution detectors, which has less efficiency and smaller field of view. Therefore, we used high efficient and large pixel size flat panel detector by choosing suitable thickness of steel wool strands and optimizing the geometrical magnification. Thanks to the geometrical magnification, fine structures in the absorption mask can be easily resolved. Here, we perform X-ray imaging on a computer memory card to demonstrate another important application for the non-destructive testing of product defects. For simplicity, we only show the retrieved transmission and dark-field image of a chip on the memory card in Fig. 3. One can immediately recognize the distinctive information unveiled by the two images. Although the subtle details for the chip are clearly displayed by the transmission image (Fig. 3a), the absorption contrast is poor in the highlighted region. The wires under the resistors are hardly noticeable (Fig. 3b), in contrast, they are clearly distinguishable in the corresponding dark-field images (Fig. 3d). Consequently, the artefact can be possibly detected from dark-field image to assess the conformity of the chip under inspection with its design, even if they are inaccessible by the conventional transmission image. Moreover, it should be mentioned that it is valuable and desirable to obtain distinct information from both absorption and dark-field imaging even if the attenuation signal is dominated by the Compton effect at higher X-ray energies^3^.

## Discussion

In summary, we have demonstrated an X-ray phase and dark-field imaging method for high X-ray energies by simply using an absorption mask consisting of steel wool, which is robust, cheap, and commercially available. Transmission image, two differential phase images and dark-field image can be produced simultaneously by scanning the absorption mask in one transverse direction only. Distinguished and complementary information is provided for complete inspection of a sample without the need to acquire any additional data acquisition. In addition to the simple experimental arrangement, the proposed technique is also compatible with the polychromatic microfocus source and high efficient flat panel detector with large pixel pitch. It should be noted that experiments can be tailored so that either large field of view or high spatial resolution can be achieved. Moreover, the data acquisition time for lab-based experiment can be further reduced by using a high brilliant metal-jet-anode microfocus X-ray tube source^17^,^18^. In addition, it is also possible to perform fast imaging with moderate angular sensitivity by further decreasing the number of images for each scan. Moreover, the proposed method can be extended to 3D high energy X-ray phase and dark-field tomography in order to visualize the internal structure of a sample^28^. If fast tomography is required, it is feasible to apply the proposed approach by using the single image speckle tracking technique with high efficiency X-ray cameras. Unlike the grating interferometer, the proposed approach can be easily adapted for either lower or higher energy region by varying the length of the steel wool strands along the X-ray beam direction. Though we have used an amber sample and a microchip to demonstrate the applicability of our technique, such a multimodal imaging method can be potentially applied to a wide range of applications involving highly absorbing materials.

## Methods

The concept of the proposed technique was first verified with synchrotron radiation source at Diamond Light Source’s B16 Test beamline. A polychromatic beam of X-rays of average energy 60 keV was filtered with 2 mm copper plate from the white beam source. In order to achieve higher absorption contrast and produce a random pattern, a bundle of steel wool (finest grade with diameter 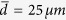) was cut to 5 mm, randomized, placed along x-ray beam direction and compressed into a polystyrene box. The distance between bending magnet X-ray source and the absorption mask was *L*_1_ = 40 *m*. A scorpion sample enclosed within manmade amber (maximum thickness is 20 mm) was placed (*L*_2_ = 0.3 *m*) downstream of the absorption mask; and the sample and detector were set to maximum achievable distance (*L*_3_ = 0.7 *m*) to maximize the angular sensitivity. The X-ray camera with an effective pixel size of 3.8 μm × 3.8 μm was based on a pco.edge detector and a microscope objective equipped with Ce-doped LuAG scintillator. A stack of 40 images was collected with and without the sample present in the X-ray beam by scanning the absorption mask vertically with a step size of μ = 2 μm, and data acquisition for each image was 1.0 s. Since the sample size (40 mm × 30 mm) was much larger than the effective field of view (4.9 mm × 4.1 mm) of the X-ray camera, 36 stacks of images were collected for the region of interest by 2D raster scanning the sample. Subsequently, transmission, horizontal and vertical differential phase images were extracted with pixel-wise analysis using a cross-correlation algorithm for each position. An advanced, phase correlation method was employed to stitch the extracted 36 images together for the four image modes^29^. The phase contrast image was then reconstructed by integrating the two stitched orthogonal differential phase images, to produce the results presented in Fig. 2.

The proposed technique was then extended to a laboratory based Nikon XT H 225 microfocus X-ray source. The X-ray tube was operated at 160 kV peak voltage and 100 μA current with average energy over 100 keV during experiment [see Supplemental Material]. Steel wool (fine grade with diameter 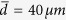) with 10 mm long strands was used in order to further increase the absorption contrast. It should be noted that the length of the steel wool can be easily adjusted (longer or shorter) to maximize the absorption contrast depending upon the average X-ray energy used; indicating that this technique can be adapted to either higher or lower X-ray regions. A tungsten reflection target was used to generate X-rays with a focal spot size of 16 μm × 16 μm, which is small to produce the magnified absorption images of the random pattern. Here, it should be noted that the focal spot size should be smaller than the diameter of steel wool in order to resolve the random absorption contrast features. The focal spot size can be further reduced by decreasing the total power of the X-ray tube, and the absorption contrast of random pattern will also be accordingly increased. Consequently, the detective angular sensitivity will be increased since the speckle tracking accuracy can be further improved with better absorption contrast of the random pattern. The images were recorded by a high dynamic range flat panel detector (Perkin Elmer 1620) with 2000 × 2000 pixels (pixel size: 200 μm × 200 μm). The exposure time for a single raw image was 250 ms. In total, 40 raw images for each scan were recorded by scanning the steel wool along vertical direction with a step size of *10* *μm*, which was the minimum step size for the existing motorized stage. The inter-distance between the source, absorption mask and the sample was set to *L*_1_ = 15 *mm* and *L*_2_ = 70 *mm*, respectively. The total distance from the source to detector was about one metre only (*L*_1_ + *L*_2_ + *L*_3_ = 1145 mm). In this study, the magnification of the sample is 16, and the effective field of view is about 25 mm × 25 mm with effective pixel size of 12 μm × 12 μm. It should be noted that either large effective field of view or higher spatial resolution can be obtained by increasing or decreasing the distance between the sample and the source. Moreover, the geometrical magnification should be optimized in such way that the fine structures in the absorption mask could be resolved by the detector.

Here we would like to emphasize that the total data acquisition time (t = 10 s) for the proposed technique is significant less than the recent reported work with a Talbot-Lau grating interferometer (t = 360 s)^9^. In addition, the power (P = 16 W) of the X-ray microfocus source is two orders of magnitude lower than the one (P = 1600 W) from a conventional X-ray tube. It should further be noted that the whole detector area can be used for the proposed technique. In contrast, only a single line pixel was illuminated due to the limited size of the gratings for each phase stepping scan by using the grating interferometer^9^.

## Additional Information

**How to cite this article**: Wang, H. *et al.* High energy X-ray phase and dark-field imaging using a random absorption mask. *Sci. Rep.* **6**, 30581; doi: 10.1038/srep30581 (2016).

## Supplementary Material

Supplementary Information

## Figures and Tables

**Figure 1:**
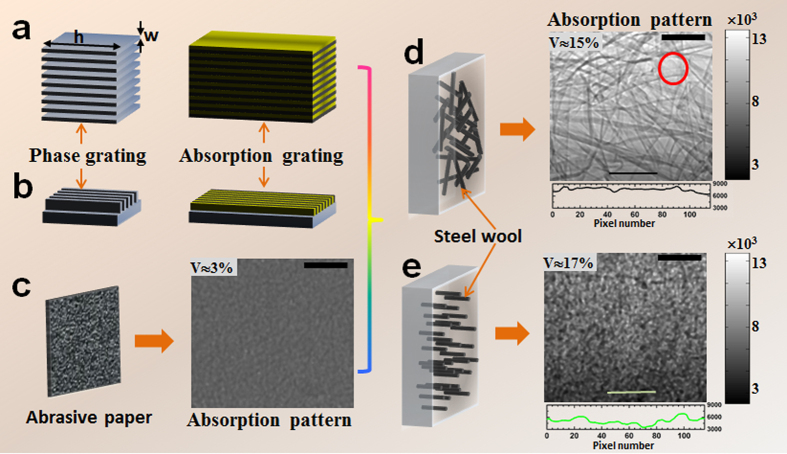
Schematic representation of the principle of grating-based, speckle-based and proposed random absorption mask based techniques for high energy X-ray imaging. (**a**) Conventional and (**b**) modified grating interferometer is based on a phase and an absorption grating. (**c**) Low speckle contrast with abrasive paper for speckle-based technique. Proposed technique with random absorption mask by arranging steel wool perpendicular (**d**) and along (**e**) the X-ray direction and higher absorption contrast random patterns were generated. The intensity profiles along the marked lines in (**d,e**)are shown at the bottom of the corresponding absorption pattern images. The scale bar is equal to 0.3 mm.
