# Supplementary material for: High energy X-ray phase and dark-field imaging using a random absorption mask
Source: Sci Rep. 2016 Jul 28;6:30581. doi: 10.1038/srep30581 (PMC4964655; doi:10.1038/srep30581)
Supplement: Supplementary Information [file srep30581-s1.pdf]

SUPPLEMENTARY INFORMATION

High energy X-ray phase and dark-field imaging using a random absorption mask

Hongchang Wang*, Yogesh Kashyap, Biao Cai and Kawal Sawhney

I. The transmission of steel wool

As described in Reference [1], the design energy is 100keV with X-ray source operated at output voltage of 160 kV with a tungsten target. In this study, the X-ray source was also operated at output voltage of 160 kV with a tungsten target for the experiment with lab-based source. When the steel wool (Fe) was inserted into the beam, it works as a filter to absorb most of lower energy radiation. The transmissions of the steel wool with different thickness are calculated as function of energy [2], and the results are shown in Fig. S1. As expected, the transmission for the lower energy region decrease with the increase of the thickness of steel wool. The total thickness of the steel wool is about 10mm in this experiment, and the effective thickness of Fe filter will be a few mm. As shown in Fig.1S, the transmission is nearly 0 for energy below 50keV when the effective thickness is 5mm, and it is only 2% at 60keV. Since most of low energy radiation from X-ray lab source has been absorbed by the steel wool, the effective working energy will be over 100keV for this study.

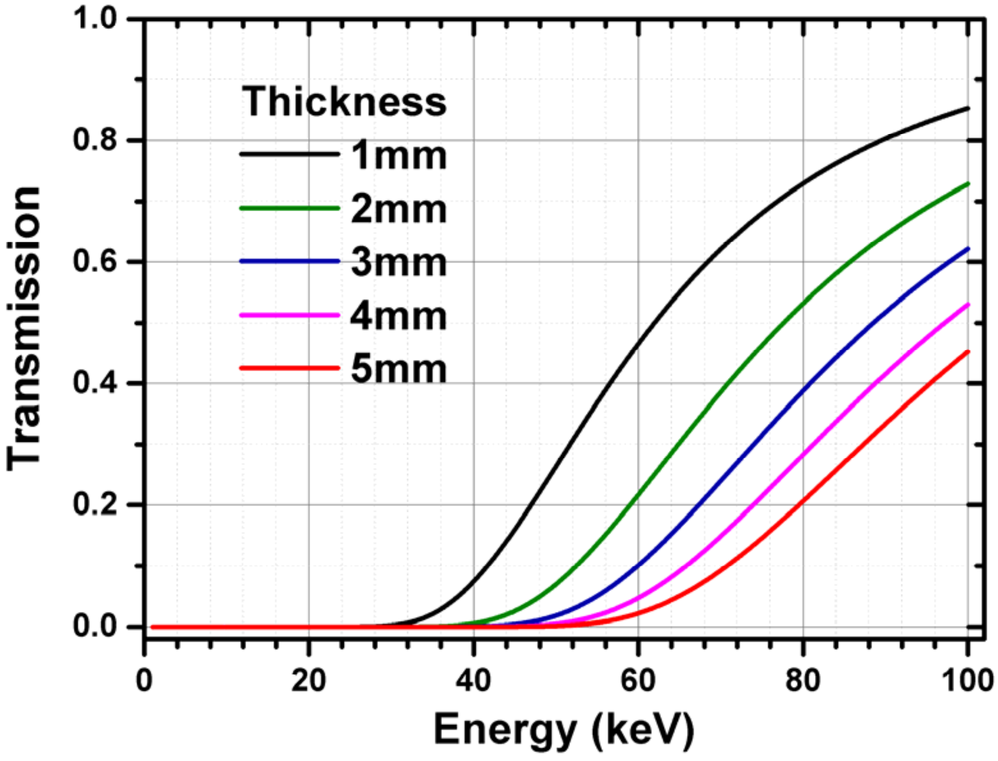

FIG. 1S. **Transmission of steel wool (Fe) as function of the energy.**

II. Calculation of transmission image from speckle scanning technique

For each detector pixel (x, y), the signal is recorded in absence f and presence g of the sample. The transmission T is then defined as the ratio of the average of two new signals f' and g', which are generated by compensating the corresponding speckle displacement [3, 4]. However, the speckle displacements at the edge of features sometime are inaccurate due to the poor correlation, and it will cause the artefacts for the derived transmission images. As shown in Fig. 2S(a), the image looks segmented at the edge of the resistors due to such artefacts. To overcome this issue, a median filter is applied to the speckle displacement maps with 3-by-3 neighbourhood. The corrected transmission image (Fig. 3b) is shown in Fig. 2s(b), and the edge artefact has been removed.

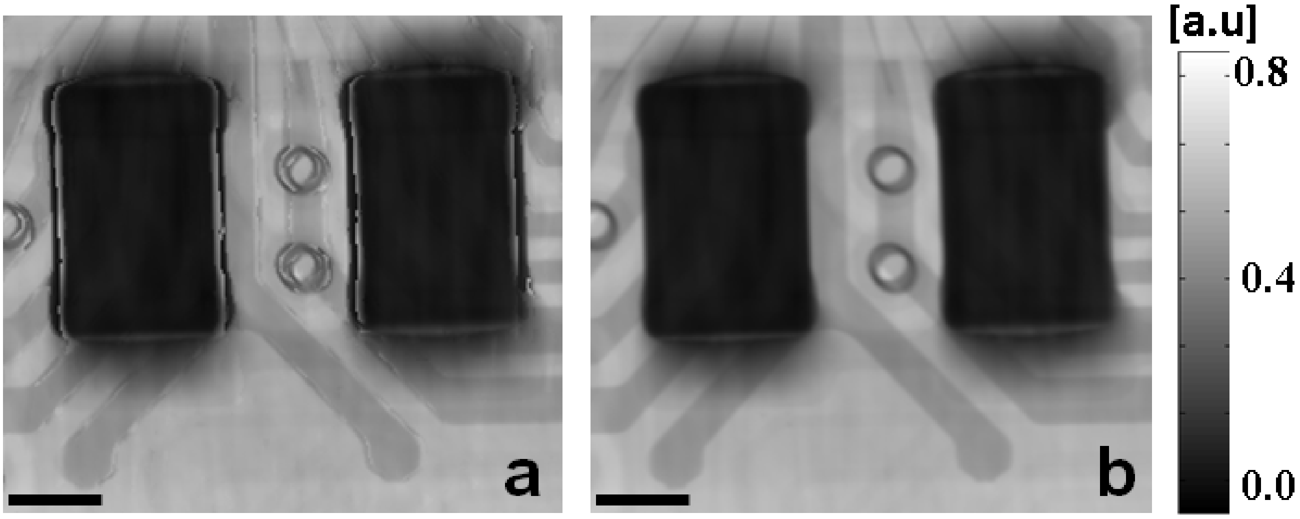

FIG. 2S. | **(a) Transmission images calculated (a) without and (b) with correction for edge artefacts**. The scale bar at the bottom left corner of each image is 0.4mm long.

----

*hongchang.wang@diamond.ac.uk

References

1. Thuring, T., Abis, M., Wang, Z., David, C. & Stampanoni, M. X-ray phase-contrast imaging at 100keV on a conventional source. *Sci. Rep.* 4, srep05198 (2014).
2. D. L. Windt, IMD - Software for modeling the optical properties of multilayer films, Computers in Physics, 12, 360 (1998).
3. Wang, H., Kashyap, Y. & Sawhney, K. From synchrotron radiation to lab source: advanced speckle-based X-ray imaging using abrasive paper. *Sci. Rep.* 6, 20476 (2016).
4. Berujon, S. & Ziegler, E. Near-field speckle-scanning-based x-ray imaging. *Phys. Rev. A* 92, 013837 (2015).
